# Supplementary material for: Identification of photoperiod-induced specific miRNAs in the adrenal glands of Sunite sheep (Ovis aries)
Source: Front Vet Sci. 2022 Jul 22;9:888207. doi: 10.3389/fvets.2022.888207 (PMC9354845; doi:10.3389/fvets.2022.888207)
Supplement: Supplementary file 1 [file Data_Sheet_1.ZIP › Supplementary file/miRDeep2 file.zip!/miRDeep2 file/Novel_Structure/SPLP42A3/Novel_191.pdf]

Provisional ID	:	NC_019475.2_29661
Score total	:	87.3
Score for star read(s)	:	3.9
Score for read counts	:	79.7
Score for mfe	:	2.2
Score for randfold	:	1.6
Score for cons. seed	:	
Total read count	:	168
Mature read count	:	153
Loop read count	:	0
Star read count	:	15

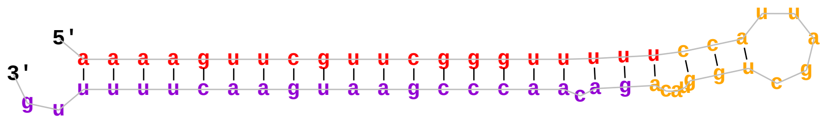

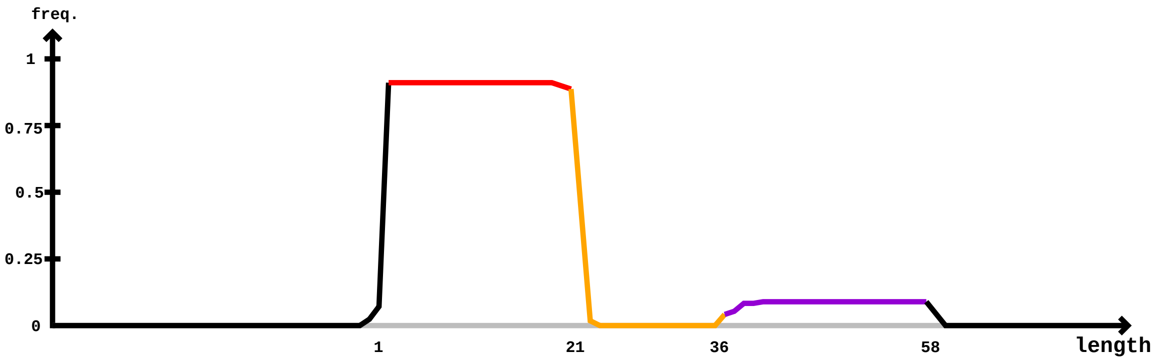


```
                                 Mature                           Star
5'- ccuggggaggaagggauguacugggguuggcccaaaaguucguucggguuuuuccauuagcugguacagacaacccgaaugaacuuuuuggccaauucaauauuuccagc   -3'   obs
    ccuggggaggaagggauguacugggguuggcccaaaaguucguucggguuuuuccauuagcugguacagacaacccgaaugaacuuuuuggccaauucaauauuuccagc         exp
    (((.....)))...(((.(((.((((((((((.(((((((((((((((((((((((.....)))...))).))))))))))))))))).)))))))))).))).)))...    reads    mm    sample
    ...............................cAaaaaguucguucggguuuu..........................................................    1        1     seq
    ...............................cAaaaaguucguucggguuuuu.........................................................    3        1     seq
    ................................caaaaguucguucggguuuu..........................................................    1        0     seq
    ................................Aaaaaguucguucggguuuuu.........................................................    5        1     seq
    ................................caaaaguucguucggguuuuuc........................................................    2        0     seq
    .................................aaaaguucguucggguuu...........................................................    2        0     seq
    .................................aaaaguucguucggguuuuu.........................................................    103      0     seq
    .................................aaaaguucguucggguuuuuc........................................................    33       0     seq
    .................................aaaaguucguucggguuuuucU.......................................................    2        1     seq
    .................................aaaaguucguucggguuuuucc.......................................................    1        0     seq
    ....................................................................gaAaacccgaaugaacuuuuug....................    6        1     seq
    ....................................................................gaAaacccgaaugaacuuuuugg...................    1        1     seq
    .....................................................................acaacccgaaugaacuuuuug....................    1        0     seq
    .....................................................................acaacccgaaugaacuuuuugg...................    1        0     seq
    ......................................................................caacccgaaugaacuuuuug....................    4        0     seq
    ......................................................................caacccgaaugaacuuuuugg...................    1        0     seq
    ........................................................................acccgaaugaacuuuuugg...................    1        0     seq
```
